# Why AgeTech Remains Invisible to Older Adults: Global Barriers and Integration Gaps

**DOI:** 10.1093/geroni/igaf122.3685

**Published:** 2025-12-31

**Authors:** Ryan Jo, Chorong Park

**Affiliations:** Elphi, West Lafayette, Indiana, United States; University of Houston, Houston, Texas, United States

## Abstract

AgeTech has surged—from remote monitoring and smart homes to wearables, safety, and social platforms—yet everyday use by older adults remains modest. We conducted a systematic scan of 300+ companies across North America, Europe, Asia, and Australia and interviewed older adults and caregivers; we triangulated findings with adoption metrics and public reviews to map barriers and enablers. Discoverability is often the first hurdle: products are hard to find until a crisis. Usability and accessibility issues persist (cognitive load, language fit, aesthetics that “signal decline”), alongside cost and reimbursement gaps, limited training or in-life support, and fragmented ecosystems that lack interoperability and electronic health record/personal health record integration. Caregiver-centric marketing sidelines older adults as primary users, reinforcing stigma, privacy and data-governance worries, and low trust. Cultural and linguistic misalignment further depresses sustained use, particularly among independently living older adults and globally diverse communities. Findings point to a practical path forward: integrated, standards-based platforms; co-created, dignity-affirming experiences; plain-language transparency and consent; funded onboarding plus ongoing support; multilingual, culturally tailored outreach; and distribution that meets people where they are (community centers, libraries, faith and cultural organizations). We conclude with procurement criteria for providers, reimbursement recommendations for training/support, and policy levers on interoperability and privacy to enable equitable, scalable AgeTech adoption—and to reframe aging as engaged, capable, and worthy of joyful technology.

